# Explainable machine learning models predicting the risk of social isolation in older adults: a prospective cohort study

**DOI:** 10.1186/s12889-025-23108-1

**Published:** 2025-05-30

**Authors:** Mingfei Jiang, Xiaoran Li, YongLu

**Affiliations:** 1 School of Public Health, Southeast University, Hunan Road, Nanjing, Jiangsu 210009 China; 2 Department of Radiology, Nanjing Gaochun People’s Hospital, No.53, Maoshan Road, Nanjing, 211300 China; 3 College of Medical Laboratory, Wannan Medical College, Wenchang Xi Road, Anhui, Wuhu 240001 China

**Keywords:** Machine learning, ML, Social isolation, Predictive modelling, Risk factors

## Abstract

**Introduction:**

This study aimed to develop a machine learning system to predict social isolation risk in older adults.

**Methods:**

Data from a sample of 6588 older adults in China were analyzed using information from China Health and Retirement Longitudinal Study from 2015 to 2018. We employed the light gradient boosting machine (Lightgbm) algorithm to determine the most common predictors of social isolation among older adults. After identifying these predictors, we trained and optimized 7 models to predict the risk of social isolation among older adults: Lightgbm, logistic regression, decision tree, support vector machine, random forest, gradient boosting decision tree (Gbdt), and Xgboost. In addition, the Shapely additive explanation (SHAP) method was used to show the contribution of each social isolation predictor to the prediction. Statistical analysis was conducted from December 2023 to April 2024.

**Results:**

The Gbdt model had the best performance with an accuracy of 0.7247, sensitivity of 0.9207, specificity of 0.6273, F1 score of 0.6894, and Area Under Curve of 0.84. In addition, the SHAP method demonstrated that intergeneration financial support, child visits, age, left-hand grip strength, and loneliness were the most important characteristics.

**Conclusions:**

The combination of Gbdt and SHAP provides a clear explanation of the factors contributing to predicting the personalized risk of social isolation for individuals and an intuitive understanding of the impact of key features.

## Introduction

As global aging accelerates, the number of elderly people is increasing, leading to a rise in the burden of disease and health disturbances [1, 2]. This is especially true in the current digital era, in which the elderly population faces not only physical and psychological health problems but also social adaptation issues and dysfunction caused by the digital divide and the prevention and control of major infectious diseases [3–5]. Social isolation has become a growing public health problem affecting the older population [6]. Studies have shown that social isolation increases the risk of many adverse health outcomes [7]. It also directly contributes to increased mortality in various disease states [8]. Therefore, accurately predicting the risk of social isolation and developing targeted interventions is critical to reducing the disease burden.

Social isolation is a condition in which an individual lacks a sense of social belonging, participation, and connectedness due to a decrease in social contacts and the size of their social network [9]. It can be measured objectively by assessing whether a person lives alone and by analyzing their social network, or subjectively through psychometric scales that assess loneliness [10, 11]. Aging can often result in a reduction in social networks because of factors such as the loss of social roles, weakened peer relationships, and death, which limit the capacity of older individuals to remain socially connected [12].

Social isolation and loneliness are weakly correlated, despite what common sense may suggest [13, 14]. Researchers suggest that social factors such as socioeconomic status and education may play a role in social isolation [15, 16]. Numerous studies have identified various risk factors for social isolation in older adults, such as age, lack of social support, intergenerational support, blood pressure, dyslipidemia, smoking, body mass index (BMI), grip strength, and depressive symptoms [17–19]. However, these studies often only examine specific predictors of social isolation rather than conducting comprehensive analyses of multiple factors. It can be difficult to reliably identify synergistic effects between variables, and researchers may have difficulty selecting which variables to include in their analysis [20]. Machine learning (ML) provides a solution to this problem by allowing for the automatic selection of variables and quantification of their statistical significance [21, 22]. Using ML algorithms, including light gradient boosting machine (Lightgbm), logistic regression, decision tree, support vector machine (SVM), random forest, gradient boosting decision tree (Gbdt), and Xgboost, we were able to construct multifactor predictive models of social isolation by analyzing many variables in exploratory and validation samples. However, existing studies mostly use cross-sectional data and usually only perform internal validation of machine learning models [23, 24]. Even studies based on cohort data are often limited to using one or two machine learning methods [25–27], which limits the generalization and applicability of the models to some extent. At the same time, it is common in existing studies to fail to fully consider sample heterogeneity, dynamic changes over time, and potential complex interactions between variables, resulting in a diminished practical application value of predictive models. Such limitations affect models’ ability to generalize across populations and scenarios and may lead to poor model performance in long-term follow-up data. In addition, the non-linear relationships of some ML algorithms make it challenging to interpret the results, which is commonly referred to as the “black box” problem [28, 29].

To address this issue, this study aimed to construct predictive models with 2-period data and compare the performance of different ML algorithms by assessing various metrics, such as accuracy, sensitivity, specificity, F1 scores, and area under the receiver operating characteristic curve (AUC). The primary goal was to evaluate the 7 models’ efficiency in predicting social isolation in China. The Shapely additive explanation (SHAP) was used to interpret individual predictions from kernel-based and tree-based models using the best-performing ML algorithm models [29]. SHAP provides a more intuitive and efficient means to visualize complex ML prediction models, which can help solve the black box problem and increase the practical application of predictive models [30].

Therefore, this study used seven ML algorithms to predict the risk of social isolation among Chinese older adults while overcoming the limitations of previous studies. In addition, the SHAP method further explained the risk of social isolation predicted by the models. The results of this study will contribute to the timely and targeted development of interventions to promote healthy aging and guide future research in this area.

## Methods

### Study population and design

**Fig. 1:**
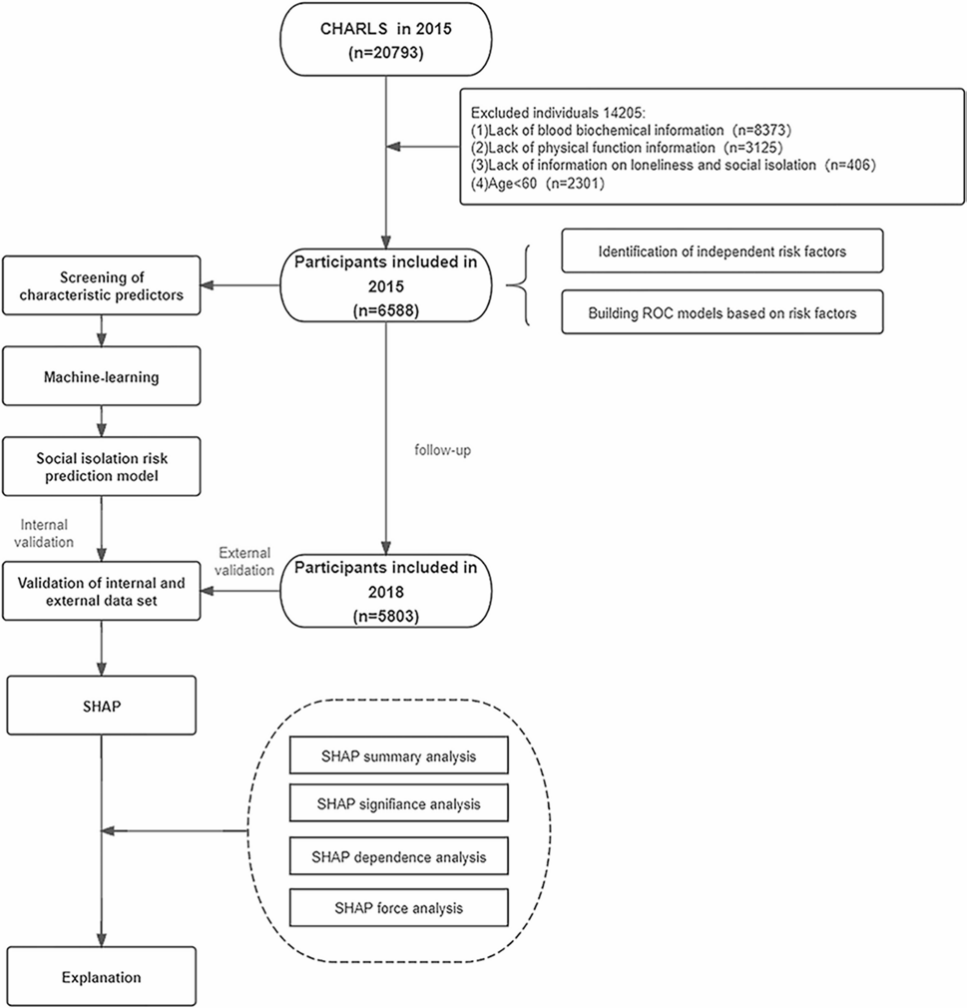
Research flowchart

This study included 20,793 investigators from China Health and Retirement Longitudinal Study (CHARLS) 2015 [31]; the data of 6588 participants were eligible for model development and internal validation. The study also included participants from the 2018 follow-up survey who were not socially isolated. Inclusion criteria were as follows: (1) participants aged 60 years or older; (2) participants with complete responses on social isolation in the 2015 and 2018 surveys; and (3) participants without a diagnosis of a serious disease such as cancer or dementia. Exclusion criteria included (1) subjects under 60 years of age; (2) lack of blood biochemical information; and (3) lack of physical function information. The study flow diagram is shown in Fig. 1. The CHARLS study received approval from the Ethics Review Board of Peking University. Informed consent forms were signed by all the respondents. The conduct of this study adhered to the guidelines set forth by the Strengthening the Reporting of Observational Studies in Epidemiology (STROBE).

### Study variables

#### Resulting variables

In the CHARLS cohort, social isolation was assessed through a self-reported questionnaire, with participants receiving a score of 1 for being unmarried (including separated, divorced, widowed, or never married), living alone, having contact with their children less than once a week (by phone, in person, or by email), and not having participated in any social activities in the past month (including interacting with friends, playing chess or cards, or attending a sport, social, or other clubs). Scores on the social isolation index ranged from 0 to 4. Participants with a score of ≥ 2 were assigned to the socially isolated group, and participants with a score of < 2 were assigned to the non-socially isolated group [6].

#### Predictive variables

An initial assessment of associated factors based on clinical significance and scientific knowledge of social isolation was conducted, and predictors were established from previous studies [14, 15]. We selected 38 factors that may be associated with social isolation: demographic characteristics, including age, sex, area, and others; laboratory results, including white blood cell and hemoglobin (HGB); physical findings, including systolic blood pressure and diastolic blood pressure; intergenerational support, including child visiting and intergenerational financial support; and other factors, including depression, loneliness, activities of daily living, and instrumental activities of daily living (IADL).

### Statistical analyses

We used independent sample *t-*tests for continuous variables and χ2 tests for categorical variables. To determine independent risk factors, we performed logistic stepwise regression analyses. We randomly assigned participants to the training and internal validation sets in a 7:3 ratio. In addition, we used the 2015 through 2018 follow-up population as the external validation set to validate the performance of the prediction model. We screened the feature predictors using Lightgbm and used fivefold cross-validation to adjust the hyperparameters. For feature importance assessment, we calculated the SHAP value for each feature and plotted the mean SHAP value to explain the effect of features on the prediction. In addition, we provided two examples of SHAP predictions for demonstration. We performed all statistical analyses using SPSS 24.0 and Python 3.10 software. Statistical analysis was conducted from December 2023 to April 2024. A statistically significant level was considered as *P* <.05.

## Results

### Baseline information on participants

Overall, 33.80% of participants (2227/6588) were classified as socially isolated, with older rural women with less education more likely to be socially isolated. Compared with non-isolated participants, socially isolated participants had higher levels of loneliness (39.07% vs. 22.47%, *P* <.001) and depression (48.72% vs. 37.26%, *P* <.001), while intergenerational support (child visiting: 52.49% vs. 83.49%, *P* <.001; intergenerational financial support: (1432.80 vs. 1851.60, *P* <.001), drinking (23.89% vs. 26.28%, *P* <.001), and surfing the Internet (0.85% vs. 3.26%, *P* <.001) were less likely. Blood biochemistry HGB (13.47 vs. 13.66, *P* <.001), HCT (40.80 vs. 41.47, *P* <.001), and TG (131.10 vs. 141.30, *P* <.001) were lower in socially isolated participants, while HDL (52.30 vs. 51.11, *P* <.001) and CYSC (0.94 vs. 0.91, *P* <.001) were higher, as shown in Table 1.

**Table 1:** Baseline characteristics of the cohort

Variable	No social isolation (*n* = 4361)	Social isolation (*n* = 2227)	t/χ2	*p*
Age(Year)	66.59 ± 5.56	69.62 ± 7.15	17.49	< 0.001
Sex(Female,%)	2184(50.08)	1163(52.22)	2.71	< 0.001
Area(Rural,%)	2606(59.76)	1545(69.38)	58.52	< 0.001
BMI(kg/m^2^)	24.38 ± 3.97	23.63 ± 3.96	7.26	< 0.001
Child visit(At least once/week,%)	3641(83.49)	1169(52.49)	718.86	< 0.001
Disabler(Yes,%)	921(21.12)	628(28.20)	41.09	< 0.001
Sleep time(Hours)	6.33 ± 1.98	6.17 ± 2.23	2.88	< 0.001
Surfing the Internet(Yes,%)	142(3.26)	19(0.85)	35.70	< 0.001
Smoking(Yes,%)	2029(46.53)	1062(47.69)	0.80	0.372
Drinking(Yes,%)	1146(26.28)	532(23.89)	4.43	0.035
ADL(Yes,%)	2138(49.03)	1087(48.81)	0.03	0.869
IADL(Yes,%)	1511(34.65)	1045(46.92)	93.56	< 0.001
Depressed(Yes,%)	1625(37.26)	1085(48.72)	79.93	< 0.001
Lonely(Yes,%)	980(22.47)	870(39.07)	201.00	< 0.001
Intergenerational financial support(Yes,%)	1851.60 ± 46.36	1432.80 ± 49.31	339.40	0.021
Sport(Yes,%)	1943(44.55)	932(41.85)	4.38	0.036
Education(High school and above,%)	3408(78.15)	1622(72.83)	23.05	< 0.001
SBP(mmHg)	139.50 ± 20.23	144.10 ± 22.26	8.44	< 0.001
DBP(mmHg)	76.17 ± 12.44	76.28 ± 12.25	0.32	0.750
Pulse(bpm)	73.71 ± 11.18	75.34 ± 11.37	5.53	< 0.001
Left-hand grip strength(lb)	27.97 ± 7.85	26.55 ± 9.05	0.98	0.329
Right-hand grip strength(lb)	28.81 ± 4.26	25.39 ± 3.07	33.68	< 0.001
WBC(10^9^/L)	5.97 ± 1.73	5.96 ± 1.92	0.05	0.963
HGB(g/dl)	13.66 ± 1.78	13.47 ± 1.94	3.88	< 0.001
HCT(%)	41.47 ± 5.39	40.80 ± 5.69	4.55	< 0.001
MCV(fl.)	92.04 ± 7.46	92.08 ± 8.49	0.16	0.869
PLT(10^9^/L)	199.50 ± 7.21	199.10 ± 7.50	2.10	0.036
TG(mg/dl)	141.30 ± 8.71	131.10 ± 8.27	45.73	< 0.001
CREA(mg/dl)	0.84 ± 0.36	0.84 ± 0.28	0.37	0.711
BUN(mg/dl)	16.11 ± 4.91	16.31 ± 5.09	1.52	0.129
HDL(mg/dl)	51.11 ± 11.78	52.30 ± 12.45	3.70	< 0.001
LDL(mg/dl)	103.70 ± 2.89	104.10 ± 2.97	5.27	< 0.001
CHO(mg/dl)	185.50 ± 3.69	185.10 ± 3.69	4.16	< 0.001
GLU(mg/dl)	105.70 ± 3.74	104.30 ± 3.44	14.76	< 0.001
UA(mg/dl)	5.06 ± 1.44	4.97 ± 1.38	2.39	0.017
CYSC(mg/dl)	0.91 ± 0.28	0.94 ± 0.26	5.58	< 0.001
CRP(mg/dl)	2.98 ± 0.64	3.00 ± 0.66	1.19	0.235
HBA1C(%)	6.09 ± 1.07	6.03 ± 0.96	2.39	0.017

Note: BMI = body mass index; ADL = Activities of Daily Living; IADL = Instrumental Activities of Daily Living; SBP = systolic blood pressure; DBP = diastolic blood pressure; WBC = white blood cell; HGB = hemoglobin; HCT = hematocrit; MCV = mean corpuscular volume; PLT = platelets; TG = triglycerides; CREA = creatinine; BUN = blood urea nitrogen; HDL = high-density lipoprotein cholesterol; LDL = low-density lipoprotein cholesterol; CHO = total cholesterol; GLU = glucose; UA = uric acid; CYSC = cystatin C; CRP = C-reactive protein; HBA1C = glycated hemoglobin

### Independent risk factors

**Fig. 2:**
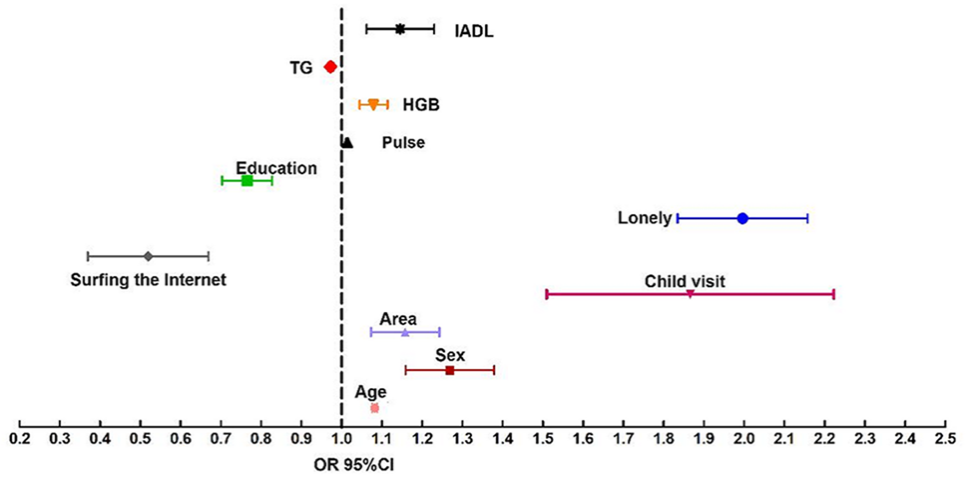
Factors associated with social isolation. IADL = activity of daily living; TG = triglycerides; HGB = hemoglobin; OR = odds ratio, represented by black graphics; 95%CI = 95% confidence interval, represented by a black horizontal line

**Fig. 3:**
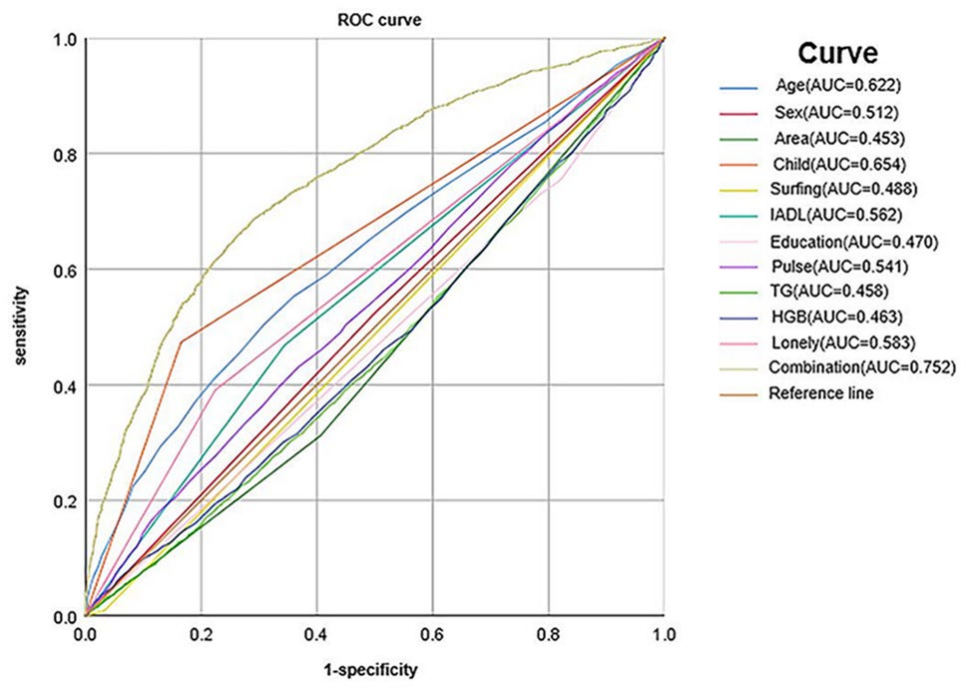
Social isolation receiver operating characteristic (ROC) curve plotted for individual or combined risk factors

We conducted a study to determine the independent risk factors associated with social isolation using the 2015 dataset. Initially, we screened 28 potential risk factors using univariate analysis, and based on the results, we identified 11 independent risk factors through logistic stepwise regression: age, sex, area, child visit, surfing the Internet, loneliness, IADL, education, pulse, HGB, and TG, as shown in Table 2; Fig. 2. Thereafter, we plotted receiver operating characteristic (ROC) curves for these factors both individually and jointly to assess their ability to predict social isolation. The combined predictive performance was found to be the best with an AUC score of 0.752, as shown in Fig. 3.

**Table 2:** Multivariate logistic Stepwise regression analysis of social isolation

Variable	β	SE.	OR	95%CI	*p*
Age	0.079	0.005	1.082	1.072–1.093	< 0.001
Sex(Female)	0.230	0.077	1.259	1.083–1.464	< 0.001
Area(Rural)	0.141	0.065	1.152	1.014–1.308	0.030
Child visit(At most once/week)	1.577	0.065	1.840	1.262–2.496	< 0.001
Surfing the Internet(Yes)	-0.742	0.263	0.476	0.284–0.797	0.005
Lonely(Yes)	0.685	0.072	1.983	1.723–2.282	< 0.001
IADL(Yes)	0.131	0.065	1.140	1.003–1.294	0.045
Education(High school and above)	-0.274	0.072	0.760	0.660–0.876	< 0.001
Pulse	0.014	0.003	1.014	1.009–1.019	< 0.001
HGB	0.075	0.029	1.078	1.020–1.140	0.008
TG	-0.002	0.001	0.971	0.962–0.985	< 0.001

Note: IADL = Instrumental Activities of Daily Living; HGB = hemoglobin; TG = triglycerides; OR = odds ratio; 95%CI = 95% confidence interval

### Development and validation of predictive models

**Fig. 4:**
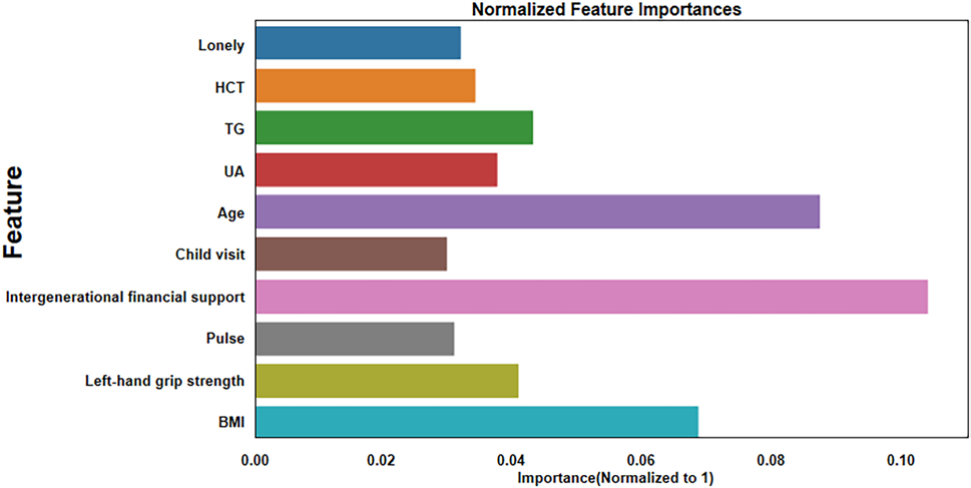
Feature selection based on Lightgbm algorithm. The horizontal axis is the importance value of each variable, and the vertical axis is the name of each variable. The bar chart displays the important values of each variable during the model calculation process. HCT = hematocrit; TG = triglycerides; UA = uric acid; BMI = body mass index

Lightgbm is a highly efficient gradient-boosting decision tree algorithm with a built-in feature importance evaluation function. Compared with Xgboost and Lasso regression, Lightgbm has a faster training speed when processing datasets with many features. To construct the social isolation risk prediction model, Lightgbm was used to normalize the 2015 training dataset, which eliminated the effect of different measurement units on independent variables. After normalization, 10 non-zero feature variables were screened and used, as shown in Fig. 4.

### Model evaluation and comparison

**Fig. 5:**
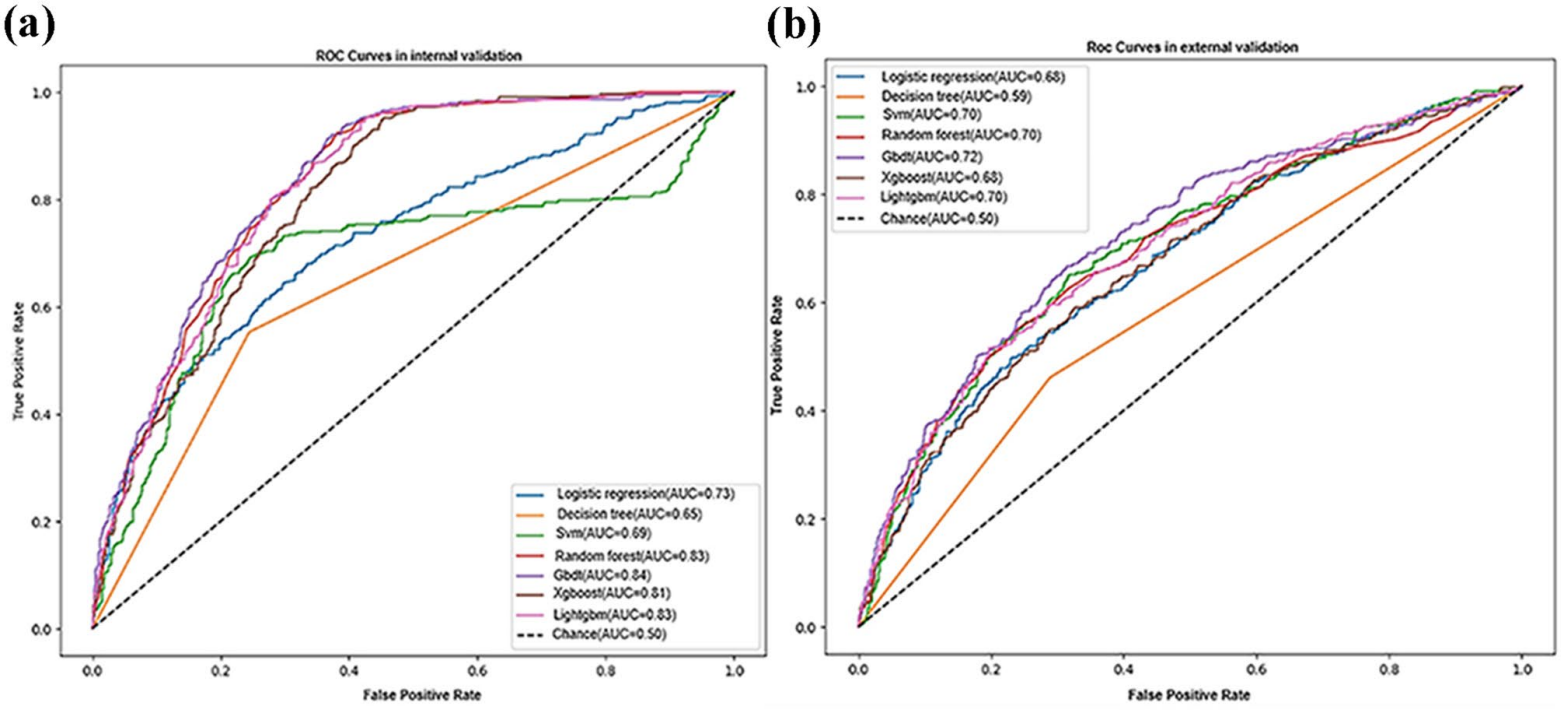
Receiver operating characteristic (ROC) curve of 7 machine learning models in internal and external validation sets. (**a**) internal validation set; (**b**) external validation set. AUC: area under the receiver operating characteristic; SVM = support vector machine; Gbdt = gradient boosting decision tree

The hyperparameters settings for the seven ML models are shown in Table 3. The results indicate that the Gbdt model demonstrated the best predictive performance on both the internal and external validation sets, with ROC curves of 0.84 and 0.72, respectively, as shown in Fig. 5. Moreover, the Gbdt model exhibited good performance according to the evaluation metrics of accuracy, sensitivity, specificity, and F1 score. We present the specific model parameters using different algorithms in Table 4.

**Table 3:** Model hyperparameters

Model	Hyperparameters	Value
logistic regression	C	1.0
	solver	‘lbfgs’
	max_iter	1000
	penalty	‘l2’
	class_weight	None
	random_state	None
decision tree	criterion	‘gini’
	splitter	‘best’
	max_depth	None
	min_samples_split	2
	min_samples_leaf	1
	random_state	None
SVC	C	1.0
	kernel	‘rbf’
	gamma	‘scale’
	probability	True
	degree	3
	random_state	None
random forest	n_estimators	100
	criterion	‘gini’
	max_depth	None
	min_samples_split	2
	min_samples_leaf	1
	bootstrap	True
	random_state	None
Gbdt	n_estimators	100
	learning_rate	0.1
	max_depth	3
	subsample	1.0
	criterion	‘friedman_mse’
	random_state	None
Xgboost	use_label_encoder	False
	eval_metric	‘logloss’
	n_estimators	100
	learning_rate	0.1
	max_depth	3
	objective	‘binary’
	random_state	0
Lightgbm	n_estimators	100
	learning_rate	0.1
	max_depth	3
	subsample	1.0
	criterion	‘friedman_mse’
	random_state	None

**Table 4:** Performance parameters of 7 machine learning prediction models in both internal and external validation sets

Predictive models	Accuracy	Sensitivity	Specificity	F-_1_ score
**Internal validation**				
Logistic regression	0.6705	0.6807	0.6655	0.5782
Decision tree	0.6752	0.5291	0.7477	0.5195
SVM	0.7340	0.6923	0.7546	0.6333
Random forest	0.7038	0.9324	0.5903	0.6762
Gbdt	0.7247	0.9207	0.6273	0.6894
Xgboost	0.6806	0.9510	0.5463	0.6640
Lightgbm	0.6984	0.9534	0.5718	0.6772
**External validation**				
Logistic regression	0.6725	0.4951	0.7715	0.5199
Decision tree	0.6181	0.4559	0.7086	0.4610
SVM	0.6716	0.6495	0.6840	0.5863
random forest	0.6163	0.7525	0.5404	0.5842
Gbdt	0.6795	0.6667	0.6867	0.5985
Xgboost	0.6444	0.5882	0.6758	0.5424
Lightgbm	0.6997	0.5147	0.8030	0.5512

### Model interpretation

**Fig. 6:**
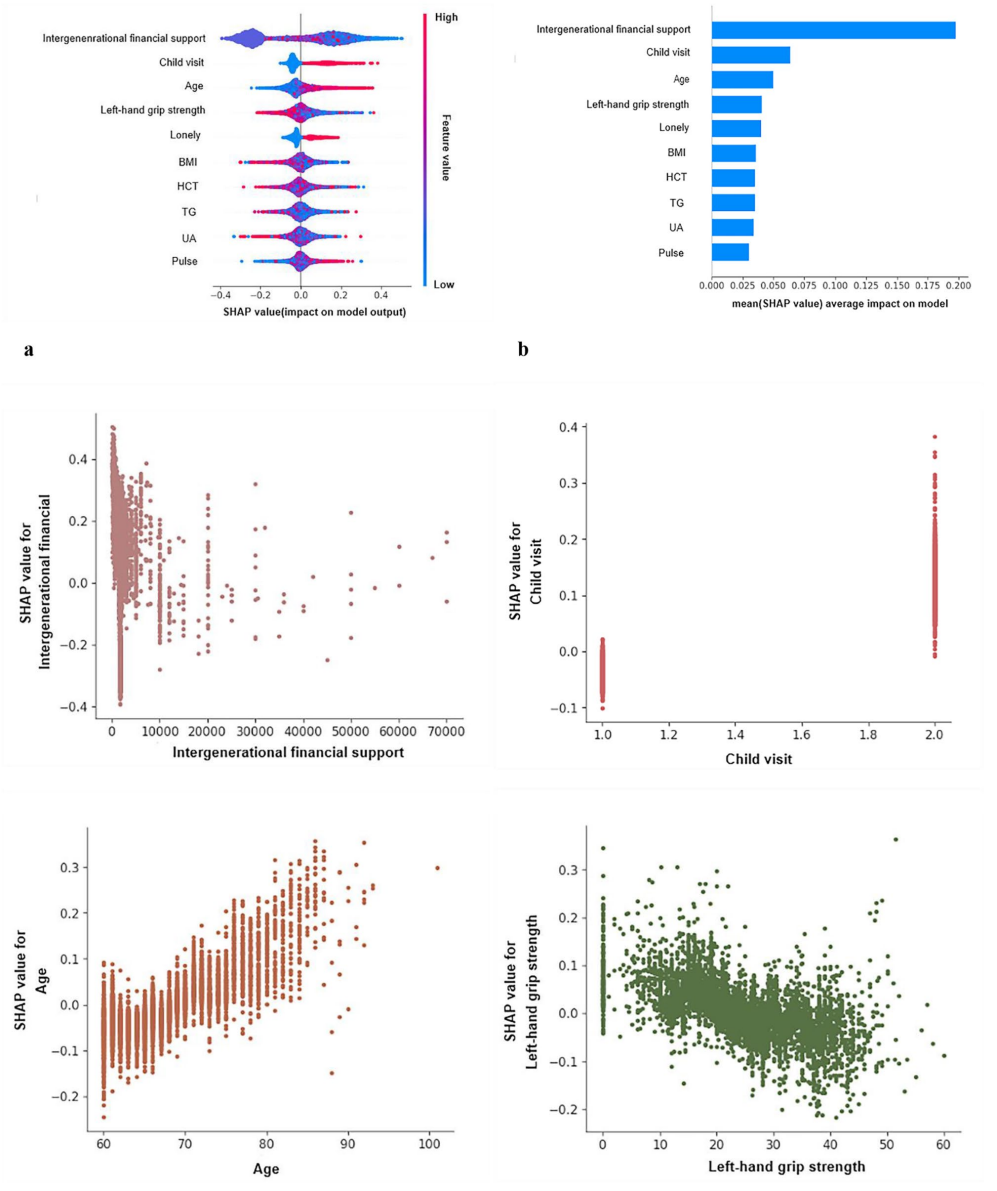

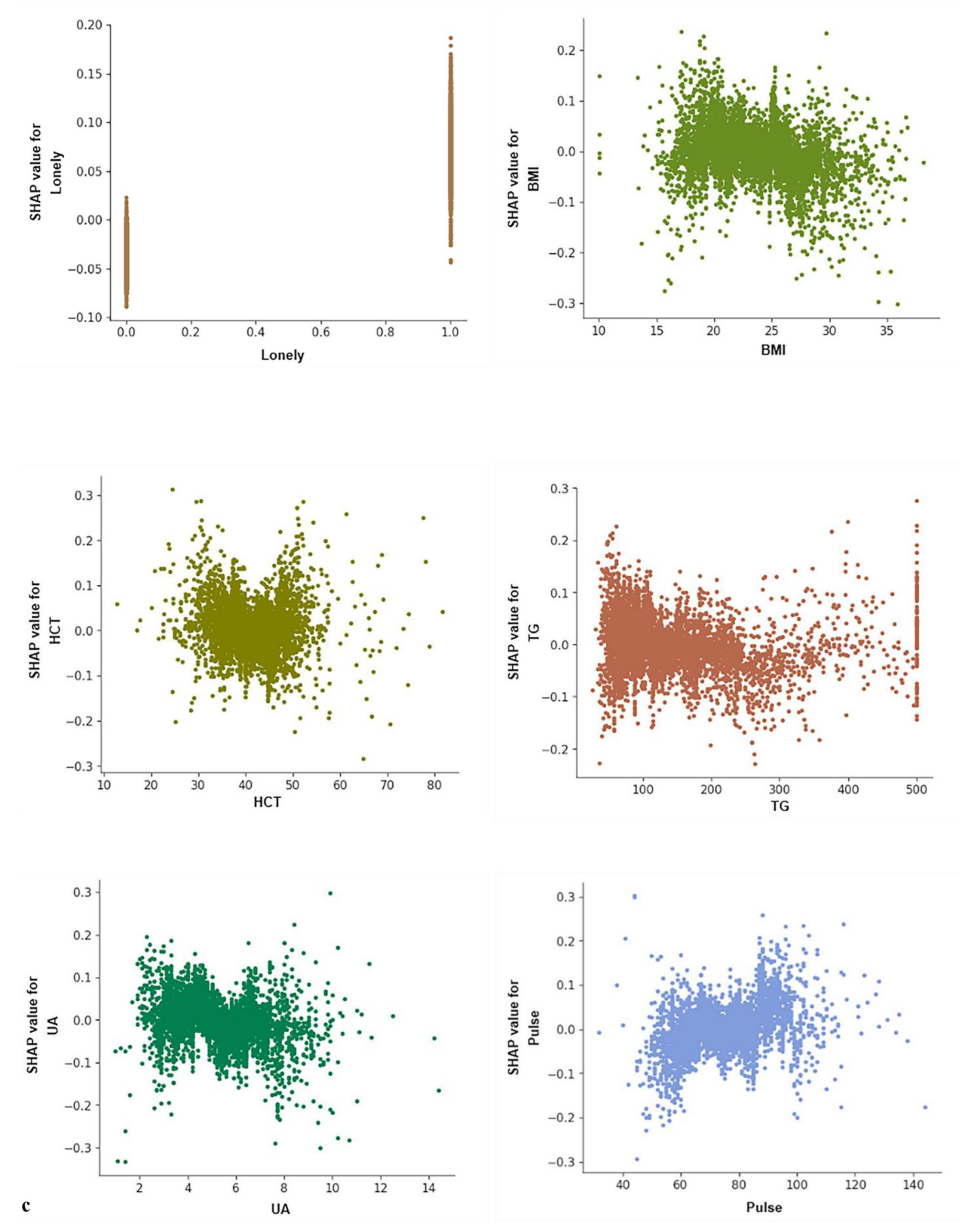

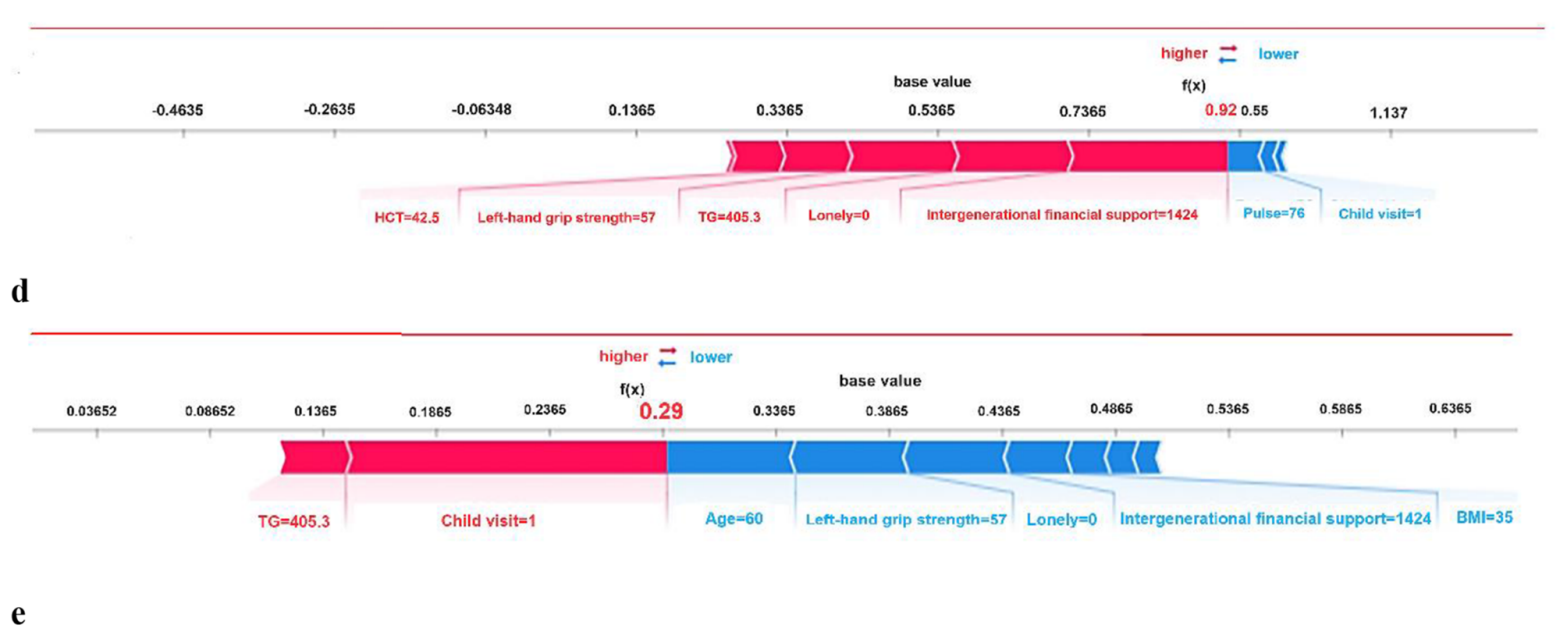
SHAP interprets the model. (**a**) All samples and features have been displayed, with each row representing a feature and the x-axis representing the SHAP value. Red dots represent higher eigenvalues, while blue purple dots represent lower eigenvalues. (**b**) Sort the importance of variables based on the average value, where the feature ranking on the y-axis represents the importance of the prediction model. (**c**) SHAP dependency diagram of Gbdt model. (**d**) SHAP prediction for the 10th sample. (**e**) SHAP prediction for the 600th sample. The red arrow indicates a higher risk of social isolation, while the blue arrow indicates a lower risk of social isolation. The length of the arrow helps to visualize the degree of influence predicted, so the longer the arrow, the more significant the impact. HCT = hematocrit; TG = triglycerides; UA = uric acid; BMI = body mass index

The SHAP values shown in Fig. 6a and b illustrate that each of the 10 features affected the average model prediction, with intergenerational support having the greatest impact. Lower intergenerational financial support and fewer child visits led to social isolation. Figure 6c shows how individual features affected the output of the Gbdt model. SHAP values tended to increase with age and decrease with increasing left-hand grip strength. In addition, we provided two typical examples to demonstrate the interpretability of the model, see Fig. 6d and e.

## Discussion

From the perspective of influencing factors, feature selection is crucial for developing predictive models. From the initial 38 variables, the Lightgbm algorithm helped identify 10 significant variables. In this study, intergenerational support emerged as a predictor of social isolation among older adults. According to some studies, receiving financial support from their children can reduce the physical dysfunction score of older individuals by approximately 0.0441 units and the depression score by about 0.1710 units. Similarly, visits from children can also help reduce the physical dysfunction score by about 0.0639 units and the depression score by about 0.0657 units [32]. Depressive symptoms include social withdrawal and negative social perceptions, and depression may exacerbate dissatisfaction with the size of social networks and their perceived quality, thereby accelerating social isolation [33]. Devine et al. found that behavioral disorders and neurobiological dysfunction may lead to social isolation and that social isolation affects physical health status in late adulthood [34, 35]. However, this study did not find depression and disability to be independent risk factors for social isolation. Age is also associated with the risk of social isolation among older people. Some research shows that the risk of social isolation increases with age and that men are particularly at a higher risk of isolation than women up to the age of 75 years [36], which is consistent with the findings of the Canadian Longitudinal Study of Aging [37]. Overweight or obese individuals may experience a lack of social connections due to social stigma, leading to wider social disconnection [38] Although this argument is convincing, it is also plausible that weight gain may serve as a potential mechanism linking perceived social isolation to poor health [39]. Furthermore, in several other studies, heavier older adults did not differ from thinner older adults in terms of perceived social support or social network size [40]. Researchers have found that reduced grip strength may be an important risk factor for social isolation in older adults, with a loss of muscle strength of 10–15% per decade after 50 years of age, accelerating to 25–40% after 70 years of age [41]. In addition, muscle mass, strength, agility, flexibility, balance, and endurance tend to decline with age, leading to reduced mobility and accelerated social isolation [42]. However, a prospective community-based study found no association between grip strength and loneliness in older participants over 80 years of age [43]. This may be because muscle strength and function decline with age and loneliness increases with age, with age-related declines in muscle strength outweighing declines in grip strength and increases in social isolation [44]. While left-hand grip strength was also associated with social isolation in this study, right-hand grip strength may not have shown a difference due to daily use habits. Indeed, social support has been associated with lower cardiovascular reactivity and ambulatory blood pressure. Similarly, immune function, cardiovascular regulation, and neuroendocrinology are negatively associated with social isolation [45]. One possible mechanism for the effect of pulse pressure on social isolation is that arterial stiffness leads to a decrease in sympathetic sensitivity, which regulates peripheral vasoconstriction, which may lead to a decrease in physical activity [46]. A large national population-based study showed that socially isolated men had lower serum uric acid levels than healthy volunteers [47, 48]. Hyperuricemia was found to be an independent risk factor for social isolation in two remote islands compared with controlled studies in coastal cities in Croatia [49]. The Baltimore Longitudinal Study of Aging found that UA and HCT were negatively associated with social participation, loneliness, and social isolation. A one-year cessation of social activities during the COVID-19 pandemic negatively affected the metabolic status of older women, particularly with significantly reduced triglyceride levels [50]. However, Cacioppo and colleagues suggested that social isolation is a potent social stressor, leading to increased cortisol and decreased glucocorticoid receptor sensitivity, increasing triglyceride levels [51]. Although current evidence supports the association of triglycerides with social isolation in older adults, the directionality of this association is unclear, and it is plausible that bidirectional effects exist. We also found that loneliness was positively associated with social isolation, which is consistent with studies such as Lu and Sung [52, 53]. In addition, Newall et al. found that social isolation and loneliness in older adults may be the same factors [54], but Hsu et al. noted that socially isolated older adults do not necessarily develop loneliness, and lonely older adults are not necessarily socially isolated [55].

Previous studies have examined various predictive models for identifying the risk of social isolation. These models often rely on Cox proportional risk models and multivariate logistic regression analysis. However, traditional statistical methods that account for the complex interplay between demographics, lifestyle, and health behaviors can be challenging to apply effectively, particularly when assuming a linear relationship between predictors and outcomes. ML surpasses traditional linear methods when modeling complex interactions and identifying non-linear patterns in data. In this study, 7 machine models were constructed, yielding AUCs ranging from 0.65 to 0.84. Despite the use of cross-validation, the Gbdt (AUC = 0.84), Lightgbm (AUC = 0.83), and random forest (AUC = 0.83) models displayed remarkably high AUC values during the modeling process and achieved high accuracy on externally validated data. Compared with classical logistic regression, the Gbdt model in this study significantly improved prediction performance (Gbdt AUC vs. combination AUC: 0.84 vs. 0.75) and achieved an accuracy of 0.7247. Unlike static, traditional statistical methods, ML can enhance performance over time through incremental learning with new data.

ML algorithms have been criticized for their lack of transparency and interpretability, making model interpretation a challenging task. To better understand the decision-making process behind these models, this study implemented SHAP scores to explain the internal logic and decision rules. The results showed a linear relationship between age and predictions effect, as is left-hand grip strength. Other factors such as TG, UA, BMI, and pulse also played a significant role in predicting the risk of social isolation in older adults, with some inflection points that require further investigation. For example, pulse initially increased the prediction of risk but then decreased slightly. This could be due to the improved health of older adults in a particular pulse group, which was not evident in the linear model. The study highlights the importance of taking individual differences into account when making predictions and emphasizes the need for personalized risk prediction. The SHAP plot provides an example of how different characteristics contribute to individual risk predictions. In the 10th prediction sample, intergenerational financial support, loneliness, left-hand grip strength, and TG were the most important factors influencing the results. This indicates the overall importance of the characteristics and reflects the heterogeneity of older adults. Overall, by analyzing SHAP scores, we can quantitatively assess the extent to which factors influence predicted outcomes, identify potential at-risk groups among older people, and provide a basis for intervention and personalized prevention.

This study has made several significant contributions to the existing literature and practice. First, we propose an interpretable machine learning model that combines the Gbdt and SHAP methods to predict the risk of social isolation among older adults. Compared to traditional statistical methods, the model improves prediction accuracy and enhances transparency and interpretability of the factors affecting social isolation, which is a significant advancement over previous studies. Specifically, this study emphasized the importance of physical indicators such as intergenerational financial support, child visits, and grip strength, which provides new perspectives for developing targeted interventions. At the practical level, children should pay more attention to emotional care while providing financial support. Regular visits, telephone contacts, and video calls can help older adults feel the warmth of family and a sense of belonging. Communities can provide a platform for older adults to communicate and interact with each other by holding activities and organizing interest groups and health talks, thus reducing their sense of loneliness. At the same time, the community should also set up specialized elderly service centers to provide health management and psychological support, as well as the early identification of individuals with declining functions and timely intervention through regular medical checkups and the promotion of physical fitness exercise programs. Policy guidance and resource support are systematic programs to address the social isolation of older adults. The Government should introduce policies encouraging intergenerational support to promote closer ties between children and older persons. At the same time, the Government should pay greater attention to the mental health, support health promotion programs through special funding, provide psychological counseling services, and disseminate knowledge about healthy aging.

Of course, there are limitations to this study: firstly, the use of a self-administered questionnaire to collect data on smoking, drinking, and health-related variables may have led to erroneous estimates; secondly, the selected variables were limited by the structure of the questionnaire; therefore, we cannot guarantee that all potential factors were included in this study; finally, the predictive model was constructed using nationally representative data, which may limit its applicability and usefulness in specific regions.

In the future, our study will further refine the data collection methods to reduce the reliance on self-reported data, especially in the collection of health-related variables such as smoking and alcohol consumption, and to improve the accuracy of the predictive models with the help of more objective health measures. Second, the study will delve deeper into the details of these predictors, for example, further analyzing the qualitative effects of financial support or exploring the psychological mechanisms between grip strength and social isolation. Meanwhile, expanding the model’s applicable population and geographic scope will help enhance its generalizability and validity in different contexts.

## Conclusion

The overall results show that the Gbdt model has the highest predictive performance and proves to be the most suitable model for identifying the risk of social isolation among older adults. SHAP analyses showed that intergenerational financial support, child visit, age, left hand grip strength, loneliness, BMI, HCT, TG, UA and pulse are the influencing factors of social isolation among older adults in China.

## Data Availability

The data that support the findings of this study are available from the Institute of Social Science Survey, Peking University, Beijing, China (http://charls.pku.edu.cn).
